# Music alters heart rate and psychological responses but not muscle activation during light-intensity isometric exercise

**DOI:** 10.1016/j.smhs.2024.01.008

**Published:** 2024-02-01

**Authors:** Andrew R. Moore, Jasmin C. Hutchinson, Christa Winter, Paul C. Dalton, Lori A. Bolgla, Vincent J. Paolone

**Affiliations:** a Department of Kinesiology, Augusta University, 1120 15^th^ St. Augusta, GA, 30912, USA; b Department of Exercise Science & Athletic Training, Springfield College, 263 Alden St., Springfield, MA, 01109, USA; c Department of Psychology, Springfield College, 263 Alden St., Springfield, MA, 01109, USA; d Heart & Vascular Institute, Penn State College of Medicine, Penn State University, 500 HMC Crescent Road, North Lobby Hershey, PA, 17033, USA; e Department of Physical Therapy, Augusta University, 1120 15^th^ St. Augusta, GA, 30912, USA

**Keywords:** Electromyography, Attentional focus, Rating of perceived exertion, Elbow flexion

## Abstract

Listening to music manipulates attention to be more externally focused, which has the potential to improve muscular efficiency. This study aimed to determine the effect of listening to music on muscle activation during an isometric exercise task, and compare this effect to those of other attentional focus conditions. Apparently healthy subjects (*n* = ​35; 16 men/19 women) completed an isometric elbow flexion task for 1 ​min in three randomized and counterbalanced conditions: internal focus (INT), external focus with a simple distraction task (EXT), or listening to music (MUS). Muscle activation of the biceps and triceps brachii and heart rate (HR) were recorded throughout the exercise tasks. Ratings of perceived exertion (RPE), affective valence, and motivation were measured at the end of each trial. There was no difference in muscle activation measures among the three conditions. HR during MUS was lower than EXT at 15 ​s ([89.4 ​± ​11.8] beats/min vs. [93.1 ​± ​12.9] beats/min; *p* ​= ​0.018) and 30 ​s ([90.6 ​± ​12.4] beats/min vs. [94.2 ​± ​12.5] beats/min; *p* ​= ​0.026), and lower than INT at 60 ​s ([93.3 ​± ​13.3] beats/min vs. [96.7 ​± ​12.0] beats/min; *p* ​= ​0.016). Overall RPE was higher for INT (13.4 ​± ​2.2) than for MUS ([12.6 ​± ​2.0]; *p* ​= ​0.020) and EXT ([11.94 ​± ​2.22]; *p* ​< ​0.001). Affective valence was higher for MUS than for INT ([2.7 ​± ​1.4] vs. [2.1 ​± ​1.5]; *p* ​= ​0.011). Manipulating attentional focus did not alter muscle activation for a light-intensity isometric muscular endurance task, though MUS was reported as more positive and requiring less exertion to complete than INT. Using music can therefore be recommended during light-intensity isometric exercise based on the psychological benefits observed.

## Abbreviations list

RPE: rating of perceived exertion
EMG: electromyography/electromyographic
HR: heart rate
BMI: body mass index
IRB: Institutional Review Board
INT: internal focus condition
EXT: simple distraction condition
MUS: music condition
PAR-Q: Physical Activity Readiness Questionnaire
3 RM: 3 repetition maximum
MVIC: maximal voluntary isometric contraction
Bpm: beats per minute
RMS: root-mean-squared
BI: activation of the biceps brachii muscle
TRI: activation of the triceps brachii muscle
CCN: cocontraction ratio of BI to TRI
*f*MRI: functional magnetic resonance imaging
*n*: numbers
*M*: mean
*SD*: standard deviation
min: Minutes
h: Hours
s: Seconds

## 1 Introduction

Continued improvements in sport and exercise performance are often sought through the use of psychological strategies.^1^ One such technique is that of directed attentional focus. The attentional focus of an individual can be defined according to the nature of the source (internal or external) of a stimulus which engages cognitive resources.^2^ One notable effect of external attentional focus on exercise performance is a decrease in the conscious awareness of fatigue. The parallel processing theory pioneered by Rejeski^3^ states that there is a limit to the amount of information that can be processed in a given instance. Sources of information simultaneously compete for the finite attentional capacity in the brain, with some information overshadowed or “drowned out” by more intense informational signals. During exercise at low to moderate intensities, the diversion of focus from internal stimuli (such as muscle exertion) to external ones (surrounding environment, conversation, etc.) limits the interpretation of afferent signals of perceived exertion (measured as rating of perceived exertion; RPE) that would manifest as the sensation of fatigue.^3^ The reduction in RPE with an external attentional focus likely results in an enhanced work capacity, due to the fact that RPE moderates central motor drive and dictates the exercise termination point.^4^ In addition to reducing the interpretation and perception of fatigue, changes in attentional focus can improve the efficiency of muscular contractions. Focusing on external cues related to performance (e.g., the result of movement on an object) rather than internal cues (e.g., the angle of elbow flexion at the time of a release of an object) during tasks of varying complexity and intensity result in improved performance outcomes, despite less muscular activation.^5^, ^6^, ^7^

The relationship between attentional focus and muscular efficiency is described by the constrained action hypothesis.^2^ It is predicted that an external attentional focus promotes the use of more automatic control processes, which increases movement fluidity and promotes more efficient motor unit recruitment. In contrast, an internal attentional focus is characterized by conscious decision-making that constrains the motor system and results in “choppy” movement patterns that are less efficient.^2^^,^^6^ During moderate intensity dynamic exercise, small changes in efficiency amount to significant changes in performance, likely by reducing unnecessary strain on the musculoskeletal system and lowering the metabolic cost of exercise.^8^ Specific to resistance exercise tasks, enhanced automaticity reduces the need for excessive joint stability,^9^ and results in a lowered metabolic cost of the task, as indicated by lowered electromyographic (EMG) activity.^9^^,^^10^ Such improvements in efficiency would likely result in a lowered exertion for a given task due to reduced peripheral fatigue and lowered central motor drive.^11^ An adaptable method of manipulating attention to be more externally focused would be a valuable tool to enhance movement efficiency.

One of the most practical and effective means to manipulate attentional focus is music. In particular, music with fast^12^ and loud^13^ qualities is capable of generating an external shift in attention. The positive effects of music have been reported during a variety of exercise protocols.^12^ Exercise at a fixed intensity is perceived to be easier when accompanied by music and is characterized by reduced physiological strain.^12^^,^^14^ Self-paced studies, in which work output is free to vary at the will of the exerciser, have demonstrated ergogenic effects.^15^ Subjects can complete a greater amount of physical work in a set amount of time while listening to music when compared to the same task under control conditions.^16^ Interestingly, an increase in work output (running speed and distance) in a finite time has been observed with music despite no difference in exercise heart rate (HR) when compared to a control condition.^17^ Performing a greater amount of work at a higher intensity without a corresponding increase in physiological strain is an additional benefit of music which warrants its use as an exercise performance strategy. The ergogenic benefits of music have also been identified during exercise trials to exhaustion, as evidenced by a significant increase in exercise time at a set intensity.^18^, ^19^, ^20^

Physiological and performance improvements are complemented by psychological benefits when music is used during exercise. These include more positive mood,^21^^,^^22^ greater task motivation,^23^ and increased exercise enjoyment.^24^^,^^25^ This enhanced psychological response is thought to contribute to the observed improvements in performance with music.^23^ Creating a more positive experience with music also has the obvious advantage of making the exercise session more pleasurable which may, in turn, promote exercise program adherence and improve long-term health.^23^ As a source of external focus, music may reduce the interpretation of fatigue and lower the metabolic cost of an activity, while also inducing psychological benefits that make exercise more enjoyable.^10^ The addition of psychological benefits makes listening to music a more attractive strategy of attentional manipulation than simply focusing explicitly on external stimuli. While the majority of music-related research has focused on continuous aerobic exercise, several studies have demonstrated the same effects in strength tasks,^26^^,^^27^ including isometric exercise. For example, listening to music has been credited with facilitating improvements in affective valence and more external thoughts, leading to greater time to exhaustion during an isometric ankle dorsiflexion task.^28^

Despite the ample evidence of improvements in physiological and psychological outcomes when listening to music during exercise, very little has been reported on the specific effect of music on muscle activation. One study indicated that music attenuates neuromuscular fatigue as measured by the electromyographic fatigue threshold during incremental single-leg knee-extensor task.^29^ However, it should be noted that the sample size of this study was small (*n* ​= ​10) and a power analysis was not reported. An internal focus of attention during isometric tasks is expected to disrupt efficient motor control, resulting in increased cocontraction of the antagonist muscle.^5^ Nevertheless, a study reported no difference in cocontraction ratio of muscles during an isometric task to exhaustion with music relative to a control (silence) condition.^28^ The fact that this exercise task was carried out until exhaustion may have obscured any difference in muscle activation. To the authors’ best knowledge, the effects of music on muscular activation have not been evaluated during a discrete task with a known end point. Moreover, the effects of music in this type of exercise task have not been compared to effects of other attentional focus manipulations.

The primary purpose of the current study was to examine differences in muscular activation during isometric exercise under different attentional focus conditions: an internal focus condition, a simple distraction task condition, and a music condition. The secondary purpose was to evaluate the psychological benefits of music by comparing RPE, affective valence (i.e., pleasure-displeasure), and state motivation among the three attentional focus conditions. It was hypothesized that muscular activation would be improved (i.e., lower) during both a simple distraction and a music condition when compared to the internal focus condition. Further, it was hypothesized that the psychological response would be most positive in the music condition, when compared to the simple distraction and internal focus conditions, resulting in lower RPE, more positive affective valence, and greater motivation for the task. HR was collected as a measure of cardiovascular demand during the trials and compared across the different attentional focus conditions. Given a lack of prior research in isometric tasks, HR was considered an exploratory variable and thus no *a priori* hypothesis was set for this outcome.

## 2 Materials & methods

### 2.1 Ethical approval

Data was collected from two different locations with the same equipment and methods utilized at each one. All methods and procedures were reviewed and approved by the Institutional Review Boards of Springfield College (IRB #156-1617) and Augusta University (IRB #1440829) prior to any data collection at the respective site. All components of the research process at both research locations adhered to the ethical standards as laid down in the 1964 Declaration of Helsinki and its later amendments or comparable ethical standards. Specifically, adherence to the US Department of Health and Human Services federal regulation 45 CFR 46 was maintained for all research conducted for this study. Informed consent was obtained from each subject prior to their participation in the study.

### 2.2 Experimental approach to the problem

The aim of this study was to examine the effect of manipulated attentional focus conditions on muscle activation and psychological response using a repeated-measures crossover design. Subjects completed a standardized 1-minute (min) isometric elbow flexion task three times under the following conditions: internal focus (INT); simple distraction (EXT); and music (MUS). Condition order was randomized and counterbalanced. Muscle activation of the biceps brachii and triceps brachii were collected during each trial. HR was collected as a measure of cardiovascular demand during the trials and to infer neuromuscular efficiency, since the same amount of work performed with a higher or lower HR may be indicative of altered neuromuscular function. Measures of RPE, affective valence, and state motivation were collected immediately following each trial to assess psychological responses to exercise during each attentional focus condition. Attentional focus ratings were also collected at the end of each trial to ensure that each condition had successfully manipulated attentional focus as intended.

### 2.3 Subjects

The program G∗Power version 3.1 was used to determine the appropriate sample size needed to detect a significant effect of condition on muscle activation.^30^ Assuming an alpha level of 0.05, a power level of 0.90, and a moderate effect size (*f* ​= ​0.25) based on a similar research design,^29^ an estimated 30 subjects would be required. Additional subjects were recruited to account for potential subject dropout and data loss. Ultimately, a total of 35 apparently healthy men (*n* ​= ​16) and women (*n* ​= ​19) between the ages of 18 and 40 years were recruited to participate in this study. All subjects gave written informed consent and completed a modified Physical Activity Readiness Questionnaire (PAR-Q) before being eligible to participate. Any “YES” response to a question on the PAR-Q excluded potential subjects from participation in the study. Demographic information for all subjects is presented in Table 1.

**Table 1:** Subject demographic information.

Variables	Men (*n* ​= ​16)		Women (*n* ​= ​19)		Total (*n* ​= ​35)	
	*M*	*SD*	*M*	*SD*	*M*	*SD*
Age (years)	22.88	(3.28)	22.21	(2.32)	22.51	(2.78)
Height (cm)	179.10	(8.08)	165.29	(8.65)	171.60	(10.82)
Weight (kg)	88.61	(18.50)	63.15	(7.74)	74.79	(18.66)
BMI (kg·m^−2^)	27.64	(5.54)	23.16	(2.77)	25.21	(4.77)

BMI ​= ​body mass index; *M* ​= ​mean; *SD* ​= ​standard deviation.

### 2.4 Testing instruments

Differential EMG sensors (DELSYS, Natick, MA) placed on the belly of the dominant arm biceps brachii and triceps brachii muscles were used to measure muscle activation. A Bagnoli 4-channel EMG machine (DELSYS, Natick, MA) recorded each muscle's electrical activity during the isometric elbow flexion trials. EMG data were sampled at 1 000 ​Hz. Subjects wore a Polar FT7 monitor (Polar Electro, Lake Success, NY) throughout the experimental session to measure resting HR and HR during the exercise trials.

A dumbbell representing ∼40% of hammer curl 3 repetition maximum (3 RM) for each individual subject (rounded to the nearest 2.5 lb) was used to standardize intensity of all trials. A plastic goniometer was used to measure the joint angle of the elbow throughout the isometric elbow task. An Apple iPod and Philips© active noise canceling headphones were used to play music for subjects during the music trial.

Measurement scales were administered following each 1 ​min trial. The Borg RPE scale^31^ was used to measure perceived exertion, the subjective rating of work intensity. Strong correlations between RPE and objective measures such as heart rate, core body temperature, and $\dot{V}O_{2}$ demonstrate a high degree of criterion validity for the Borg RPE scale.^32^^,^^33^ Test-retest reliability of RPE has also been established, using a running task on separate days.^34^ Affective valence was assessed with the Feeling Scale,^35^ a single-item scale with possible responses ranging from −5 (*very bad*) to +5 (*very good*). An attentional focus scale^36^ was used to assess subjects’ attentional focus (internal or external) during exercise. The scale ranged from 0 to 100, with 0 representing a completely external focus of attention and 100 representing a completely internal focus of attention. Finally, a single-item scale was used to measure state motivation.^37^ The scale ranged from 0 (*Not at all motivated*) to 10 (*Extremely motivated*). There is a strong rationale for the applicability of single-item scales, as long as they demonstrate high face validity.^38^ Instructions for all four scales were phrased in the past tense, as they were used following completion of exercise trials, rather than during trials.

### 2.5 Experimental conditions

During INT, subjects were asked to look at the biceps of the dominant arm, and focus all attention on the contracted biceps and resulting sensation throughout the 1 ​min trial. The researcher gave an attentional focus reminder prompt (“Look at your arm and focus on the feeling in the muscle”) to subjects every 15 ​seconds (s) following HR collection, to ensure adherence to the condition instructions.^10^ During EXT, subjects were asked to look at, and read aloud, a series of numbers displayed on a printed piece of paper throughout the 1 ​min trial. The paper consisted of seven lines of 12 numerals in size 70 font and was taped to the wall ∼2 ​ft from the subjects' face. Subjects read aloud the first three-digit number starting with the digit in the top left corner of the page, then did the same for the three-digit number starting with the subsequent digit, and so on until the end of the trial. This task was designed to occupy attention with a basic process while limiting cognitive stress. During MUS, subjects listened to music they selected from a list of seven possible tracks. The tracks were selected by the experimenter to represent a variety of genres, and all were characterized by fast tempo of (104-122 beats per minute [bpm]) and moderate lyrical complexity. This approach is recommended in order to account for subjects’ personal preference while still standardizing critical music elements such as tempo.^39^ The music intervention was delivered at a standardized volume of 80 ​db. Subjects were instructed to focus their attention on the lyrical and rhythmical qualities of the track throughout the 1 ​min MUS trial.

### 2.6 Testing procedures

**Fig. 1:**
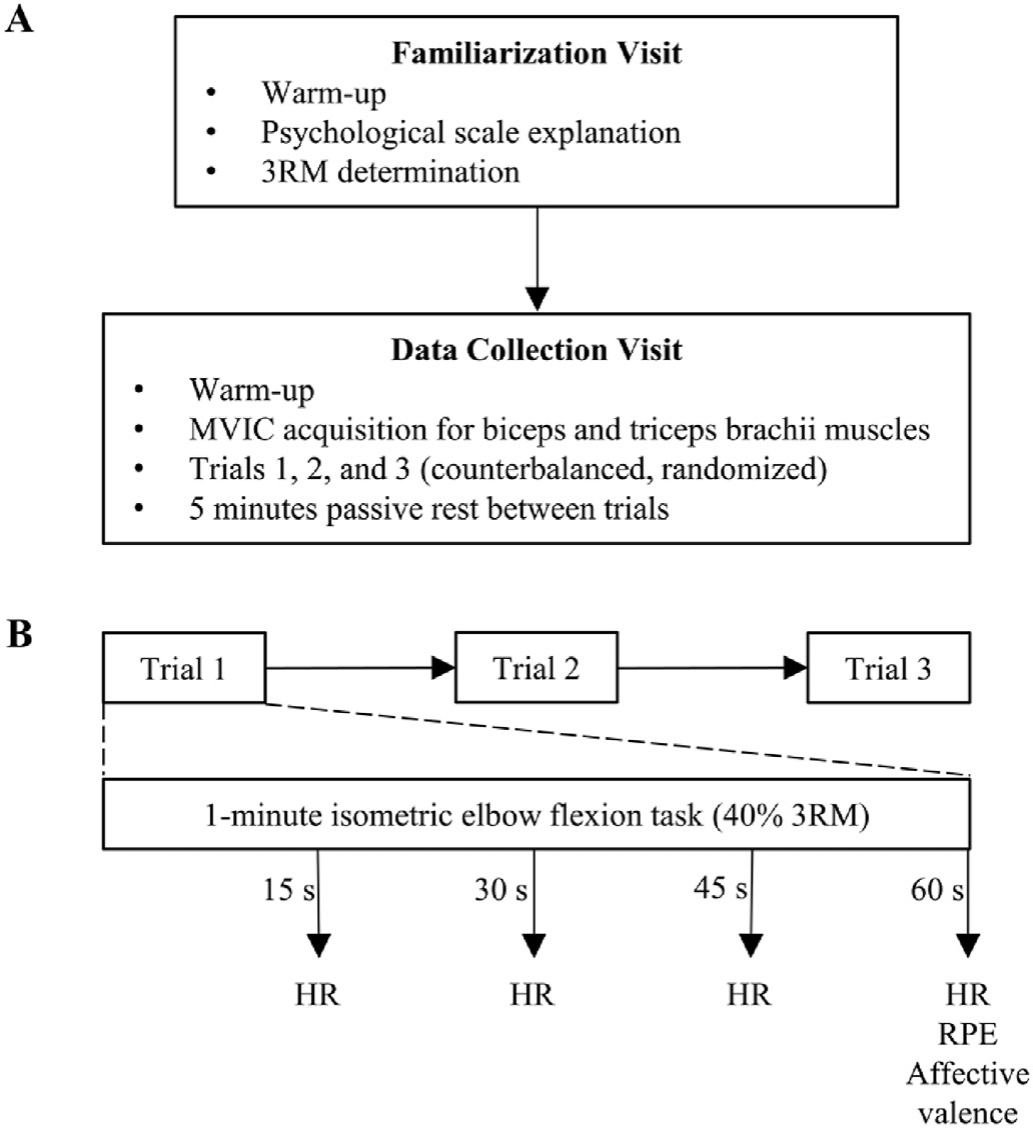
Study protocol timeline. **A** – summary of protocol events on each of the two data collection visits. **B** - summary of data collection timeline for each trial (INT – internal focus condition; EXT – simple distraction condition; MUS – music condition) of the data collection visit. Heart rate (HR) collected every 15 ​second (s); rating of perceived exertion (RPE) and affective valence collected at 60 ​s; 3 RM – 3 repetition maximum; MVIC – maximal voluntary isometric contraction.

Following completion of Informed Consent and the modified PAR-Q, eligible subjects visited the laboratory on two occasions. A visual summary of each visit and the data collection timeline is presented in Fig. 1. Subjects were instructed to abstain from alcohol, caffeine, and strenuous exercise in the 24 ​hour (h) prior to each study visit.

#### 2.6.1 Familiarization session

Subjects completed a 5 ​min walking warm-up of self-selected intensity on a treadmill. Acquisition of subject 3 RM for the dumbbell bicep hammer curl exercise followed the warm-up, using the dominant arm. Subjects completed three warm-up sets of dumbbell hammer curls, increasing from low to moderate intensity, before attempting the first of three 3 RM trials. The highest successful 3 RM with proper form was used to calculate the weight of the dumbbell used during the experimental trial (i.e., 40% of 3 RM). Criteria for proper hammer curl form included fixation of the elbow at the mid-axillary line of the thorax throughout the entire movement, alignment of the spine perpendicular to the ground to prevent compensatory leaning, and flexion of the elbow throughout the entire range of motion for each repetition. The initial session also served to familiarize subjects with the data collection process for the experimental session. Measurement instruments were reviewed, and subject understanding of the scales was ensured by prompting responses on each scale following each set of the 3 RM acquisition.

#### 2.6.2 Experimental session

At least 24 ​h following the initial familiarization session, each subject completed the three experimental trials in a single visit. Upon arriving at the laboratory, subjects completed a 5 ​min walking warm-up at a self-selected pace on a treadmill. Electrodes were then placed on the biceps and triceps muscles in accordance with established guidelines.^40^ A light musculoskeletal warm-up specific to the biceps was completed by instructing subjects to maintain two, 3 ​s contractions at 25%, 50%, and 75% of estimated maximal effort, in increasing order, with 15 ​s rest between contractions. The maximal voluntary isometric contraction (MVIC) consisted of the subjects producing a maximal contraction against an immovable object (safety rail on the power rack) for 3 ​s. Three biceps MVIC attempts were completed by each subject, with a rest period of at least 1 ​min separating attempts. Following completion of biceps warm-up and MVIC, the same procedure was followed for the triceps. The highest activation value recorded during the MVICs represented 100% activation and was used to normalize muscular activation during the experimental isometric elbow flexion tasks.

The three trials with different attentional focus instructions were administered in a randomized and counterbalanced order. During each trial, flexion of the elbow was monitored by the researcher using a goniometer attached to the subject's arm, with the fulcrum at the lateral epicondyle and the goniometer arms fixated on the lateral aspect of the radius and humerus with athletic tape. Upon deviation of > 10° of elbow joint angle from the exercise position, the researcher instructed the subjects to correct the amount of contraction to maintain 90° of flexion (“Move up” or “Move down”). Throughout all three experimental trials, electrical activity of the biceps and triceps was recorded and saved onto a computer for statistical analysis. HR was recorded every 15 ​s throughout all trials.

Immediately following each trial, psychological variables were measured by displaying the corresponding scale and reading the prompt for each of the measurement scales one at a time. Subject responses were recorded by a research assistant. Subjects were sufficiently acquainted with the measurement scales during the familiarization session and warm-up such that response generation time was limited and collection of this data took no longer than 30 ​s after the termination of each of the three trials. The rest period between each experimental trial was 5 ​min, during which subjects completed a simple math exercise as a washout task to eliminate the residual effects of any of the attentional focus manipulations.^41^ Any subject whose HR failed to return to within 5 bpm of the previously recorded resting heart rate during the rest period receive an extended passive rest until this requirement was met.

#### 2.6.3 EMG analysis

For each 1 ​min trial, EMG data were band-pass filtered between 20 ​Hz and 450 ​Hz and normalized to 100% of the MVICs completed at the beginning of the experimental session. The 1 ​min reading was divided into four 15 ​s bins to analyze the effects of contraction time on muscle activity changes. The EMG signals for each bin were converted to a root-mean-squared (RMS) value, which was then used to compare the effect of condition (INT, EXT, and MUS) on muscle activation. The RMS values of the biceps (BI) and triceps (TRI) were then used to calculate cocontraction (CCN) of the two muscles by dividing BI by TRI at each time point, in each condition.

### 2.7 Statistical analysis

Muscular activation in all trials was evaluated by comparing normalized RMS values of the muscles measured. A series of 3 ​× ​4 mixed factorial ANOVAs were performed to analyze the dependent variables BI, TRI, CCN, and HR. The first repeated-measures factor was condition, and consisted of three levels: INT, EXT, and MUS. The second repeated-measures factor was time, and consisted of four levels: 15 ​s, 30 ​s, 45 ​s, and 60 ​s. Psychological responses were compared between the three conditions using a series of one-way repeated-measures ANOVAs. The dependent variables were RPE, affective valence, and motivation. Additionally, attentional focus was analyzed as a manipulation check using a one-way repeated-measures ANOVA as described above.

All data were screened for normality and outliers. All analyses were completed using IBM SPSS version 28 with an alpha level of 0.05 for each analysis. If the assumption of sphericity was violated according to Mauchly's Test of Sphericity, the Greenhouse-Geisser value was used to make adjustments. In the case of significant main effects, Bonferroni adjusted post-hoc tests were completed. Effect size is reported as partial eta squared (*η*^*2*^), and is interpreted according to the guidelines described by Cohen (1988) as small (*η*^*2*^ ​= ​0.01), medium (*η*^*2*^ ​= ​0.06), and large (*η*^*2*^ ​= ​0.14).^42^

## 3 Results

A total of 35 subjects (16 men and 19 women) completed all three experimental trials. Complete descriptive and inferential statistical results are available at the following liked external data repository: https://osf.io/4fu69/.

There were three cases of missing data which resulted from technical difficulties. Four subjects had at least one missing HR data point and two subjects were missing all EMG data for one of the trials. The subjects with missing HR or EMG data points were removed from the respective analyses. These subjects, however, were included in all other analyses for which their data was complete. Sample size varied in different analyses for this reason, with fewer subjects in the HR and all EMG analyses than in demographic data, RPE, affective valence, motivation, and attentional focus.

### 3.1 Manipulation check

There was a significant main effect of condition on attentional focus (*F*_[2,68]_ ​= ​120.53, *p* ​< ​0.001, *η*^*2*^ ​= ​0.78) such that attentional focus was significantly more internal in INT than in EXT and MUS (*p* ​< ​0.001 for both comparisons). The difference between EXT and MUS was not significant. Therefore, attentional focus was successfully manipulated in all conditions, and MUS was an effective means of manipulating attentional focus externally.

### 3.2 EMG data

No significant interaction effects or main effect of condition were found for BI, TRI, or CCN. There was a main effect of time for BI (*F*_[1.41, 45.15]_ ​= ​34.64, *p* ​< ​0.001, *η*^*2*^ ​= ​0.520), TRI (*F*_[1.08, 34.63]_ ​= ​9.44, *p* ​= ​0.003, *η*^*2*^ ​= ​0.228), and CCN (*F*_[1.10, 35.44]_ ​= ​6.12, *p* ​= ​0.016, *η*^*2*^ ​= ​0.161). BI was significantly lower at 15 ​s than at all other time points (*p* ​< ​0.001 for all) and significantly lower at 30 ​s than at 45 ​s (*p* ​= ​0.006) and 60 ​s (*p* ​= ​0.003). TRI was significantly lower at 15 ​s than at 30 ​s (*p* ​< ​0.001), 45 ​s (*p* ​= ​0.008), and 60 ​s (*p* ​= ​0.022). CCN was significantly lower at 15 ​s than at 30 ​s (*p* ​= ​0.008). All other post hoc comparisons were not significant. Mean and standard deviation values are presented in Table 2.

**Table 2:** Mean and standard deviation values for EMG (electromyography) variables and heart rate in three different conditions at four time points.

Variables	time points	INT		EXT		MUS	
		*M*	*SD*	*M*	*SD*	*M*	*SD*
BI	15 ​s	22.89	(11.73)	21.83	(10.71)	22.08	(11.60)
	30 ​s	25.48	(12.22)	24.67	(11.25)	25.09	(11.75)
	45 ​s	27.05	(13.34)	25.73	(10.55)	26.06	(11.57)
	60 ​s	27.62	(12.35)	26.47	(10.87)	26.91	(11.35)
TRI	15 ​s	12.25	(7.15)	12.02	(7.46)	12.18	(7.86)
	30 ​s	12.48	(7.12)	12.15	(7.46)	12.39	(7.86)
	45 ​s	12.65	(7.10)	12.23	(7.55)	12.49	(7.85)
	60 ​s	12.65	(7.21)	12.30	(7.59)	12.62	(7.80)
CCN	15 ​s	2.98	(2.60)	2.90	(2.38)	3.27	(3.57)
	30 ​s	3.18	(2.71)	3.27	(2.77)	3.60	(3.78)
	45 ​s	3.34	(3.12)	3.53	(3.28)	3.73	(4.16)
	60 ​s	3.38	(2.93)	3.66	(3.50)	3.84	(4.38)
HR	15 ​s	91.09	(13.83)	93.12	(12.90)	89.39	(11.77)
	30 ​s	91.61	(13.24)	94.24	(12.51)	90.61	(12.37)
	45 ​s	94.18	(11.92)	94.55	(12.84)	92.33	(12.70)
	60 ​s	96.67	(11.97)	94.73	(13.20)	93.30	(13.27)

*M* ​= ​mean; *SD* ​= ​standard deviation; INT ​= ​internal focus condition; EXT ​= ​simple distraction condition; MUS ​= ​music condition; BI ​= ​biceps brachii % activation; TRI ​= ​triceps brachii % activation; CCN ​= ​cocontraction ratio of BI to TRI; HR ​= ​heart rate.

### 3.3 HR data

**Fig. 2:**
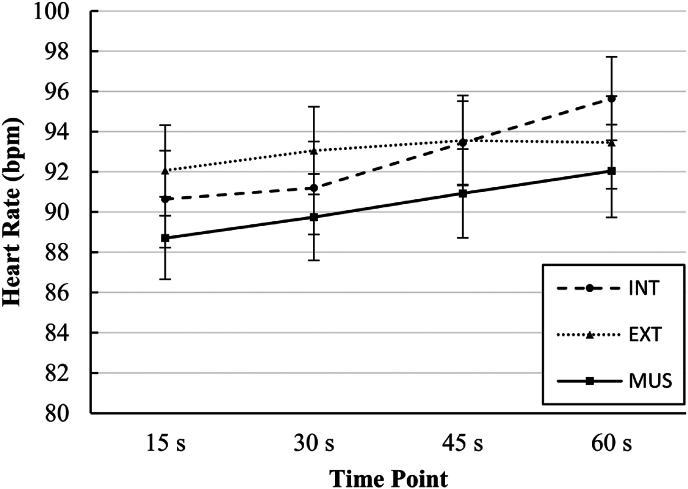
Heart rate at different time points (15, 30, 45, and 60 ​seconds [s]) in different conditions INT ​= ​internal focus condition; EXT ​= ​simple distraction condition; MUS ​= ​music condition; bpm ​= ​beats per minute. Markers represent mean values and whiskers represent standard error about the mean values.

There was a significant two-way interaction between time and condition for HR (*F*_[4.06, 129.82]_ ​= ​4.54, *p* ​= ​0.002, *η*^2^ ​= ​0.124). HR was significantly lower in MUS than EXT at 15 ​s (*p* ​= ​0.018) and 30 ​s (*p* ​= ​0.026) and significantly lower than INT at 60 ​s (*p* ​= ​0.016). There was also a main effect of condition on HR (*F*_[2, 64]_ ​= ​3.65, *p* ​= ​0.032, *η*^*2*^ ​= ​0.102), though none of the group differences were significant following Bonferroni adjusted comparisons. An expected significant main effect of time on HR (*F*_[1.57, 49.82]_ ​= ​12.86, *p* ​< ​0.001, *η*^*2*^ ​= ​0.287) was observed, but was not interpreted given the significant interaction effect.^43^ All other interaction effects and main effects were not statistically significant. Mean and standard deviation values are presented in Table 2. Mean values and the nature of the interaction effect are displayed graphically in Fig. 2.

### 3.4 Psychological data

**Fig. 3:**
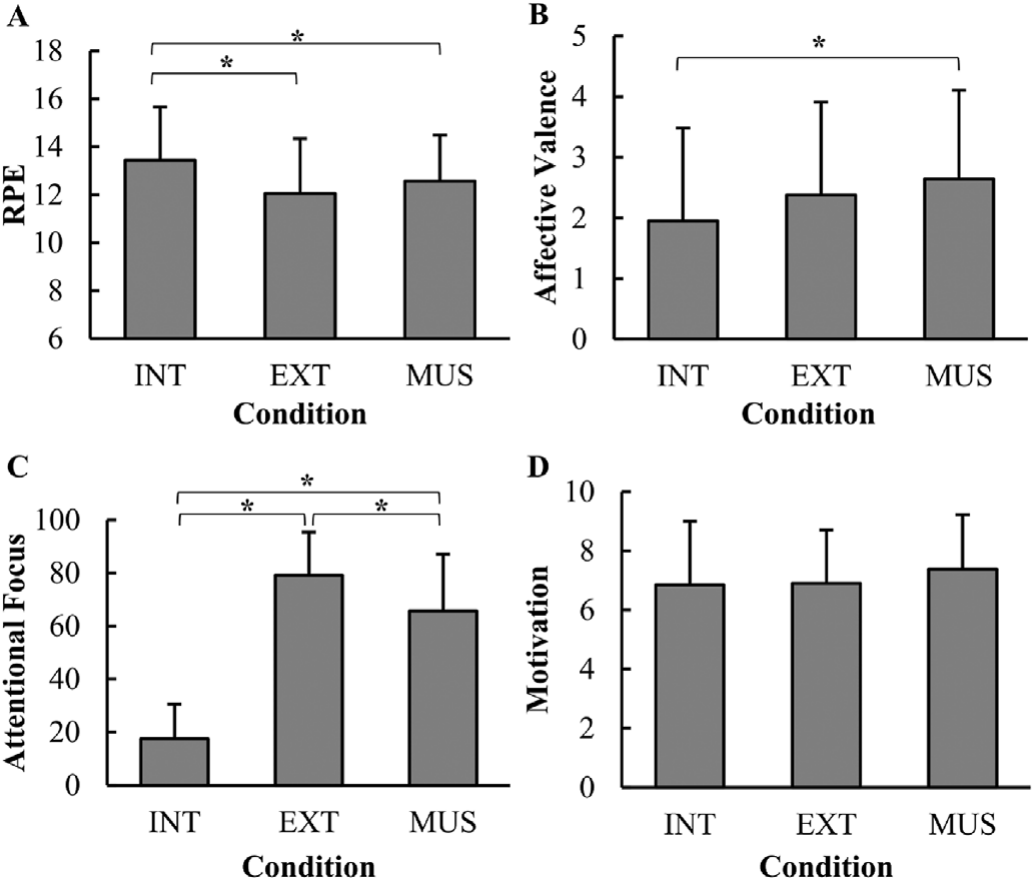
Psychological responses to exercise in different conditions. INT ​= ​internal focus condition; EXT ​= ​simple distraction condition; MUS ​= ​music condition; (A) Rating of perceived exertion (RPE); (B) Affective valence; (C) Attentional focus; and (D) State motivation. ∗ ​= ​significant main effect between conditions (*p* ​< ​0.05). Bars represent mean values and whiskers represent standard deviations from the mean.

There was a significant effect of condition on RPE (*F*_[2,68]_ ​= ​10.28, *p* ​< ​0.001, *η*^*2*^ ​= ​0.232) and affective valence (*F*_[2, 68]_ ​= ​3.79, *p* ​= ​0.028, *η*^*2*^ ​= ​0.100), but not motivation (*F*_[2, 68]_ ​= ​1.01, *p* ​= ​0.371, *η*^*2*^ ​= ​0.029). RPE was significantly higher for INT than for EXT (*p* ​< ​0.001) and MUS (*p* ​= ​0.020). Affective valence was significantly higher (more positive) in MUS than in INT (*p* ​= ​0.022). All remaining post hoc comparisons were not significant. Mean and standard deviation values are presented in Table 3 and displayed graphically in Fig. 3.

**Table 3:** Mean and standard deviation values for psychological measures for three different conditions.

psychological measures	INT		EXT		MUS	
	*M*	*SD*	*M*	*SD*	*M*	*SD*
Attentional focus	17.80	(12.75)	77.86	(16.73)	66.63	(20.46)
RPE	13.43	(2.16)	11.94	(2.22)	12.63	(1.99)
Affective valence	2.09	(1.52)	2.51	(1.54)	2.69	(1.41)
Motivation	6.89	(2.11)	7.03	(1.79)	7.31	(1.84)

INT ​= ​internal focus condition; EXT ​= ​simple distraction condition; MUS ​= ​music condition; *M* ​= ​mean; *SD* ​= ​standard deviation.

## 4 Discussion

The current study was designed to determine the effects of different types of attentional focus manipulations (internal focus, external focus using a simple distraction task, and external focus using music) on muscle activation and psychological responses during an isometric elbow flexion task. The main finding was that attentional focus condition did not impact muscle activation during the 1 ​min task, though perceptual and psychological responses were more favorable as a result of using music.

No differences in muscle activation of the biceps or triceps brachii muscles were observed between conditions. These findings are in agreement with other researchers who reported no differences in cocontraction between internal and external focus (music) conditions during ankle dorsiflexion at 40% MVIC.^28^ Authors of a different study reported that there was a lower cocontraction ratio during 1 ​min ankle plantar flexion when external attentional focus was utilized.^5^ It is difficult to determine the precise reason for differing results between reports. However, the improvements in motor function observed with an external focus appear to be more consistent and dramatic during dynamic exercise tasks than during isometric, single-joint tasks.^2^ Dynamic tasks require a greater amount of coordination and motor control than do isometric contractions. As such, there may be a greater potential for internal focus to serve as a hindrance to exercise, in accordance with the constrained action hypothesis.^2^

The utilization of an overly simple isometric form of exercise with a small muscle mass in the current study may have accounted for the lack of differences in EMG values observed between attentional focus conditions. The use of music may therefore be more appropriate for exercise using more complex movements requiring a higher intensity and degree of coordination.

Muscle activation was the primary outcome of this study, though HR was also assessed as an exploratory variable to measure cardiovascular demand during the trials. No *a priori* hypothesis was set for HR though, interestingly, it was lower in the MUS condition at the beginning and end of the trials relative to EXT and INT, respectively. This suggests that listening to music is associated with reduced physiological strain, which is consistent with prior works.^12^^,^^14^

One potential mechanism of action for the reduction in HR we observed with MUS is the effect of music on entrainment or synchronization of breathing, because breathing frequency modulates nervous system function.^44^^,^^45^ Conscious or subconscious respiratory synchronization with the auditory structure of the music may focus breathing in a way that promotes parasympathetic nervous system activation, thus reducing HR slightly. This would explain why HR was lower at certain time points for MUS compared to both EXT and INT despite the same muscle activation and work intensity.

Although the differences in HR in this experiment present little practical value (< 4 bpm), this observed effect is more meaningful when interpreted in the context of the exercise challenge. Due to the short duration, low intensity, and small amount of active muscle mass during each experimental trial, a relatively small HR response (an increase of ∼22 bpm above resting HR) was elicited. If a similar decrease in HR relative to the cardiovascular challenge (∼18%) was observed during more demanding exercise, the effect of music on HR may be more robust and have more meaningful implications for performance.

Evidence of the effect of directed attentional focus (i.e., INT vs. EXT) on HR during isometric exercise is lacking, thus our current findings make a novel contribution. We observed no significant difference in HR between INT and EXT conditions. Prior research using aerobic exercise also reported no differences in HR when running under different focus conditions, despite a significantly lower $\dot{V}O_{2}$ during running with a directed external focus.^10^ The results from the current study support this finding, as HR was lower only in the MUS, but not the EXT condition, relative to INT. The use of music as a source of external attentional focus, rather than directed attention, may contribute to decreases in cardiovascular strain during physical activity.

Differences in cardiovascular or neuromuscular strain may be accompanied by changes in psychological variables. We observed that RPE was improved in both EXT and MUS conditions compared to INT. Reductions in RPE are frequently reported with an external attentional focus. One explanation for this decreased perception of effort is the theory of parallel processing established by Rejeski.^3^ Signals of fatigue and physiological strain are thought to determine the conscious perception of effort, but only when appropriate attention is afforded to process these signals in the sensory areas of the brain. Distribution of limited attentional resources to other stimuli (external environment, music, etc.) limits the attention garnered by fatigue, and hence reduces RPE. This theory is supported by *f*MRI (functional magnetic resonance imaging) research in which areas of the brain are non-invasively scanned to assess activity of neurons. During exercise, the addition of music was found to cause increased activation of a region that is inversely correlated with perceived exertion.^46^ Music may interfere with the processing of signals of exertion, thus inducing an ergogenic effect. The results from the current study support this idea, as the same relative amount of muscle activation and work performed in all conditions led to different perceptions of effort that were lower in the MUS trial. RPE was also lower in EXT than in INT, though it is difficult to say if simple distraction impacts the same brain region implicated in past research since the study authors did not include such a trial in their study.^46^

Along with an improvement in RPE, MUS was characterized by a more positive affective valence when compared to INT. Increases in exercise pleasure and enjoyment with the addition of music are widely documented.^12^^,^^39^ The results from this study align with past reports by demonstrating a psychological benefit of listening to music even during exercise of light intensity and short duration with a small muscle mass. Isometric exercises are widely used in rehabilitation settings, injury prevention programs, and in preparatory exercises for athletes.^47^ Isometric exercise is an effective form of exercise during immobilization following injury or surgery and has been associated with enhanced strength and range of motion.^48^ Given that commonly reported barriers to rehabilitation exercises include pain/discomfort and lack of enjoyment,^49^^,^^50^ music may offer an engaging and enjoyable stimulus that can promote adherence to an exercise rehabilitation program.^51^ There were no differences in motivation between conditions, perhaps due to the low physical requirement of the exercise task. A more vigorous or prolonged exercise task would have challenged the motivational state of subjects to a higher degree, presenting a greater potential for the beneficial motivational effects of music or other external focus that have been reported elsewhere.

A limitation of the current study was the type of exercise task selected. The most notable improvements in motor function and cardiovascular demand with attentional focus manipulation are observed during multi-joint dynamic exercise. Isometric elbow flexion was selected to minimize EMG noise artifact and obtain the most valid measurements of muscular activation, with the tradeoff making it more difficult to observe an effect on muscle activation. A second limitation was the intensity of the exercise task. In an effort to complete all three conditions on the same visit while also limiting the effects of residual local muscle fatigue between trials, a light intensity was selected for the task (40% of 3 RM). Using a heavier weight may have made detection of neuromuscular differences easier.

## 5 Conclusions conclusion

The attentional focus conditions used in this study (internal focus, external focus, and music) did not alter muscle activation for a light-intensity isometric muscular endurance task. However, the use of music during the task enhanced affective valence and decreased perceived exertion compared to the internal focus condition despite the same work performance and muscular activation in both conditions. The use of music can therefore be recommended during isometric exercise of light intensity based on the psychological and perceptual benefits observed. Physical and occupational therapists, organizational psychologists, and other practitioners who prescribe sustained light-intensity exercise may find these results relevant and applicable.

## Submission statement

All authors have read and agree with the manuscript content. The manuscript will not be submitted elsewhere for review while it is being reviewed for publication in Sports Medicine and Health Science.

## Ethical approval statement

Data was collected from two different locations with the same equipment and methods utilized at each one. All methods and procedures were reviewed and approved by the Institutional Review Boards of Springfield College (IRB #156-1617) and Augusta University (IRB #1440829) prior to any data collection at the respective site. All components of the research process at both research locations adhered to the ethical standards as laid down in the 1964 Declaration of Helsinki and its later amendments or comparable ethical standards. Specifically, adherence to the US Department of Health and Human Services federal regulation 45 CFR 46 was maintained for all research conducted for this study. Informed consent was obtained from each subject prior to their participation in the study.

## Authors' contribution statement

**Andrew R. Moore:** Writing – review & editing, Writing – original draft, Methodology, Investigation, Formal analysis, Conceptualization. **Jasmin C. Hutchinson:** Writing – review & editing, Writing – original draft, Methodology, Conceptualization. **Christa Winter:** Writing – review & editing, Formal analysis. **Paul C. Dalton:** Writing – review & editing, Investigation. **Lori A. Bolgla:** Writing – review & editing, Investigation. **Vincent J. Paolone:** Writing – review & editing, Supervision, Project administration.

## Conflict of interest

The authors declare that they have no known competing financial interests or personal relationships that could have appeared to influence the work reported in this paper.
